# Assessment of intellectual disability in Klinefelter’s Syndrome associated with other Prenatal and Perinatal risk factors. A case report

**DOI:** 10.1192/j.eurpsy.2022.1561

**Published:** 2022-09-01

**Authors:** T. Castellanos Villaverde, A. Hospital Moreno, I. Louzao Rojas, E. Fernández-Jiménez

**Affiliations:** Hospital Universitario La Paz, Department Of Psychiatry, Clinical Psychology And Mental Health, Madrid, Spain

**Keywords:** Klinefelter syndrome, prenatal and perinatal difficulties, intellectual disability, nonverbal intelligence

## Abstract

**Introduction:**

Klinefelter’s syndrome (KS) is considered as a genetic risk factor for intellectual disability and specifically for impairment on verbal skills. Prenatal and perinatal morbidity concurrent with this diagnosis determine the entity and typology of neurodevelopmental deficits.

**Objectives:**

To describe the intellectual and adaptive functioning of a patient with KS and prenatal and perinatal difficulties, assessed in the Service of Psychiatry, Clinical Psychology and Mental Health at La Paz University Hospital (Madrid).

**Methods:**

A descriptive study was conducted of a 6 year and 7-month-old boy with diagnosis of KS (karyotype XXY), extremely low weight at birth (752 gr.), Arnold Chiari type I malformation with post-hemorrhagic hydrocephalus and periventricular leukomalacia, bronchopulmonary dysplasia, intra-atrial septal defect, left kidney agenesis with iatrogenic adrenal insufficiency, and acute ventriculitis due to E. Coli. Neuropsychological evaluation was carried out in October 2021. Leiter-3 scale was selected due to the absence of expressive language; and his parents completed the questionnaire Adaptive Behavior Assessment System-II.

**Results:**

The nonverbal intelligence quotient was observed in the very low range (full-scale IQ = 69). The result of general adaptive behavior (z = -2.83), showed very high functional disability both in the conceptual, social, and practical domains.

**Conclusions:**

The high number of causes of disability in this patient is consistent with a high degree of functional disability. Efficient evaluation sessions of intellectual performance, adaptive functioning, and necessary supports, due to the absence of expressive language and limited receptive language, are required. A specific neuropsychological evaluation profile should be established for KS.

**Disclosure:**

No significant relationships.

